# The perils of the first try: experimental evidence for visuomotor calibration in darts and hammering

**DOI:** 10.1007/s00426-025-02161-w

**Published:** 2025-07-22

**Authors:** David J. C. Smith, Philip Furley, Fabian Wunderlich, Herbert Heuer, Daniel Memmert

**Affiliations:** 1 Department of Institute of Exercise Training and Sport Informatics, German Sports University Cologne, Cologne, Germany; 2 Leibniz Research Centre for Working Environment and Human Factors, Dortmund, Germany

## Abstract

Task such as hammering or throwing darts involve intentional actions performed with the anticipation of a desired effect that requires precision to achieve success. Visual perception of the goal, defined in an external frame of reference, plays a crucial role in specifying movement parameters in a body-centered frame of reference. Physical interruption of the task decouples the internal and external frames of reference leading to rapid performance decrements. Motor calibration, as noted by the ‘Calibration Effect’, is the fine-tuning of motor commands following performance-related feedback in the external frame of reference. Here we test the calibration effect with non-skilled populations in both a sport specific and an everyday type of task, darts and hammering. Participants performed 20 rounds of five attempts of hitting a goal with a stable bodily position. Between rounds they moved around to perturb the fine tuning. The first repetition within a round of repeated attempts was less accurate than the subsequent attempts within that same round, even if controlling for gradual learning effects. Thus, the calibration effect, a rapid component of warm-up, is present both in professional athletes and unskilled dart players as well as in everyday activities such as hammering.

## Public significance statement

In any sport or activity which requires precision we depend on our perceptions that offer us key feedback on refinements and adjustments required to improve our own skills and abilities. This research examines how we rapidly refine or ‘calibrate’ our movements during a darts and hammering task to help us improve our chances for scoring points or not hammering the fingers instead of the nail.

## Introduction

Humans often seem to struggle on the first try of a series of performances in a wide range of tasks. There are likely numerous explanations for this phenomenon such as a lack of practice or unfamiliarity. However, even highly skilled and expert individuals have been shown to perform less accurately on the first trial in a series of performances than in subsequent ones (Wunderlich et al., 2020; Phatak et al., 2020). This phenomenon has been termed the calibration effect. This effect has been observed in few retrospective performance analyses of high-level sport competitions that involve highly precise perceptual-motor skills and within experimental studies (e.g. van Beers, 2009). In such studies, the visuomotor calibration was explored utilizing dart players to understand the cognitive process of the phenomenon itself. That is, van Beers (2009) studies “how our brain uses errors in previous movements to adjust planning of future movements” as it pertains to variability within the task itself. Such research helps establish the calibration effect but is limited in focus on the variability of repetitions and error correction within a set of dart throws. Hence, the goal of the present research was to investigate the visuomotor calibration across a series of sets in a laboratory paradigm involving hammering and throwing darts to further explore the reset and repeatability of visuomotor calibration when the relation between the internal frame of reference for motor control and the external frame of reference for target location is perturbed (Adams, 1961; Brand & de Oliveira, 2017; Fajen, 2005b; Gibson, 1979; Heuer & Massen, 2013; van Andel et al., 2017; van Beers, 2009; Warren, 1984).

A task such as hammering or darts involves an intentional action with a desired effect, namely, to hit the nail and/or target. While in hammering the tool remains in physical contact with the body whereas in darts the dart is thrown, both require similar motor movements and are essentially ballistic, which is why they were selected. According to Wunderlich et al. (2020), the production of an intentional movement involves a series of transformations. These “motor transformations” are between neural signals that begin at a cortical level and end with action potentials on the muscle fibers, followed by muscle contractions to produce movements that serve to accomplish the desired task, which often is defined by certain perceived changes in the environment. Identification of the neural commands at the start of this series of transformations that at least approximate the intended outcome requires the inversion of the transformations, that is, the determination of the input given the desired output. This can be achieved in two primary ways, the first being based upon visual feedback and the second being based on an internal model of the transformations (Admiraal et al., 2004; Ajemian et al., 2010; Lackner & DiZio, 2000; Sülzenbrück & Heuer, 2011; van Andel et al., 2017; van Beers, 2009; Wunderlich et al., 2020).

Tasks such as throwing darts or hammering are too rapid for continuous control based on visual feedback. Thus, they have to rely on internal models of the motor transformations, and only the error visible after each attempt can be used to fine-tune them. Internal models represent a complex set of internal calculations to specify movement parameters that respect the motor transformations relevant for the task. Task relevance allows for variability to adapt to the tools and objects being used for the task, which harkens again back to the childhood development of basic motor movements developed early on (Heuer & Massen, 2013). Be it throwing a dart, throwing a ball, throwing an axe, throwing mom’s car keys, the same basic motor transformations occur with adaptions relative to the object’s unique characteristics (Heuer & Massen, 2013; Fajen, 2005a, b; Fajen, 2005b; Lackner & DiZio, 2000; Phatak et al., 2020; van Beers, 2009; Wunderlich et al., 2020).

Both hammering and darts typically involve a repeated number of trials, (e.g., three throws in darts based on the game rules, however many swings it takes to hammer a nail into a board). This repeated sequence of sensorimotor actions in both tasks is physically interrupted between series of trials, such as retrieving the darts after your turn or adding a new nail. Arguably, this interruption could lead to performance variations between the individual repetitions of the task, particularly between the first and second repetition, but also amongst the others. In particular, the interruption can result in a loss of calibration which could be re-established based on the visual feedback after the first repetition so that the second repetition is more likely to hit the target (Phatak et al., 2020; van Beers, 2009; Wunderlich et al., 2020). This has recently been coined the calibration effect (van Beers, 2009; Phatak et al., 2020; Wunderlich et al., 2020) and is consistent every time the body-related frame of reference has been briefly decoupled from its external counterpart. Most likely the calibration effect is a component of the established ‘warm-up effect’ which has been noted in the performance of fine motor skills as it relates to expertise and the amount of focused, intentional practice both in acquiring the skill and immediately prior to the performance itself (Ajemian et al., 2010; Phatak et al., 2020; Wunderlich et al., 2020). Both the warm-up effect and the calibration effect are linked and compatible with broader theories of sensorimotor adaptation, motor control, and motor learning such as error-based learning (cf., Redding & Wallace, 2006; Schmidt & Wrisberg, 2008; van Beers, 2009). For example, van Beers (2009) explored the calibration effect in relation to the PAPC (Planned Aimed Point Correction) model of how the brain corrects for errors within a motor movement.

For a long period, the warm-up effect was explained in terms of the set hypothesis which refers to task specific adjustments in mental and physical states that may influence performance (Phatak et al., 2020; Wunderlich et al., 2020). However, the set hypothesis is limited to physically active and skilled athletes for whom such arousal states are an effect of the task itself, such as playing basketball, hockey, or football. The set hypothesis was established in motor tasks in which participants underwent physically intense activities that lasted for several minutes in duration coupled with an extended rest period. The focus of that research was on manipulating the arousal state of the body via the sympathetic nervous system through active periods of movement followed by rest. While certainly insightful, that research contrasts with throwing a set of darts or hammering a nail which typically lasts only a few seconds and doesn’t create an arousal state in the body quick enough to affect performance (Fajen, 2005b; Phatak et al., 2020; Schmidt & Nacson, 1971; Wrisberg & Anshel, 1993; Wunderlich et al., 2020).

As the set hypothesis is inadequate to account for the rapid performance decrements and increments observed in games like darts, Wunderlich et al. (2020) considered the ‘Perceptual-Motor Calibration Hypothesis’ (i.e., the calibration effect; as an extension to the cognitive calibration effect in judgment and decision making; Unkelbach et al., 2012; Unkelbach & Memmert, 2014) according to which movement parameters and thus the internal model of the motor transformations, which are defined in a body-related frame of reference, have to be calibrated with respect to an external frame of reference. Both darts and hammering involve an extension of the elbow and a flick of the wrist as a power movement timed with the release of the dart on the throw or the position of the hammer head swung onto the intended target. To refine these motor skills requires repeated practice coupled with trial and error to learn the ideal timing and coordination of the motor movement toward successfully performing the task. It is generally understood that as a result of practice, performance improves, leading skilled people to spend hours upon hours in practicing the task to refine their skill and precision and improve performance (Fajen, 2005b; Phatak et al., 2020; Wunderlich et al., 2020).

Ajemian et al. (2010) note that performance is subject to miscalibration within breaks if other activities, even as simple as walking, are performed. Visuomotor calibration is subject to ever-present “noise” within the perceptual motor system, that is the sheer number of stimuli present within one’s environment and in one’s internal thought processes. This is notably apparent in performance and can lead to drifting of the initial calibration adjustments during breaks. As it relates back to the initial notions of the warm-up decrement, the more the task is practiced and developed, the ability to focus and tune out the noise while maintaining focus for consistent performance relative to the environmental conditions gains importance (Jacobs, 2007).

Hence high skilled performers tend to see reduced performance variations following an initial warm up session (Ajemian et al., 2 010). However, Ajemian et al. (2010) specifically mention that this only applies to “highly skilled athletes”, whereas lesser skilled and inexperienced athletes may not observe such a significant performance difference regardless of warm up. Therefore, the present research addresses this shortcoming in the literature by investigating the calibration in inexperienced performers (Ranganathan et al., 2020; van Andel et al., 2017; van Beers, 2009). More specifically, the goal of this research was to test the calibration effect in non-skilled populations and extend the generalizability of the effect by testing it in both a sport specific (darts) and “everyday” type task (hammering). Generally, we expected the calibration effect to be present in non-skilled populations and to exist independent of the task. In particular, we assume that the calibration is perturbed when the body physically moves away from its position following each round, which means that the effect remains in each new round even if the movement itself has already been repeated many times. Consequently, we hypothesized that the first repetition within a round of repeated attempts from a fixed position would be less accurate than the subsequent attempts within that same round.

## Method

### Transparency and openness

This research conforms to the standards of transparency as listed in the TOP Guidelines. All data for the study was collected between the summer of 2022 and the fall of 2023. The findings of this paper are based on controlled experimental research, which limits its straightforward applicability to more complex and dynamic environments. We report how we determined our sample size, all data exclusions (if any), all manipulations, and all measures in the study.

### Power analysis

**Fig. 1:**
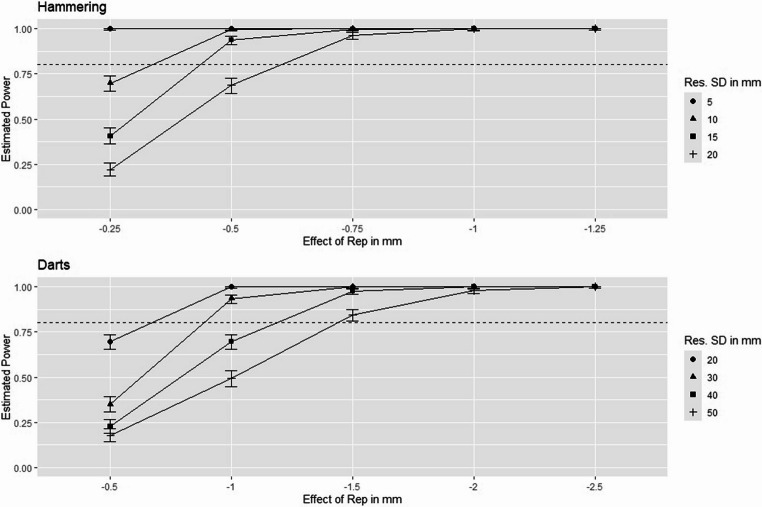
Power estimated by simulation for various scenarios. Effects of Repetition are illustrated on the x-axis and estimated power on the y-axis. Different lines and symbols represent varying residual standard deviations. Error bars refer to 95% confidence intervals. All simulations refer to a fixed number of 50 participants with 20 rounds of 5successive repetitions, which results in a total of 5 000 attempts per task

Power analyses usually calculate the minimum sample size required to reach an acceptable power based on an expected (but often somewhat speculative) effect size. We pursue a slightly different approach, where we fix the experimental design and test a variety of realistic scenarios (i.e. effects and residual variances). Through a simulation-based power analysis (Kumle et al., 2021), we obtain the power estimated from 500 simulations for each scenario using the package *simr* (Green & MacLeod, 2016) in R Statistical Software (v 4.3.1; R Core Team, 2023). The package generates artificial data with predefined properties (e.g. sample sizes, effects, variances), analyses them with the target model and estimates power by calculating the fraction of simulations that correctly reject the null hypothesis (Green & MacLeod, 2016). We chose this approach as we lacked usable effect sizes for comparable tasks reported in previous studies, which is even true for the highly related study of Wunderlich et al. (2020) being limited to experts (professional darts players) and not reporting numerical deviations. Moreover, the model complexity and number of assumptions required is increased for mixed models and established analytical solutions for power estimation may not be standardly available. The focus of our simulation is on the presence of a calibration effect being consistent with the hypothesis of the study. Please find the assumptions for the power simulations chosen in light of the experimental setups and the complexity of the tasks in Table 1 and the results in Fig. 1.

**Table 1:** Assumptions taken for the power simulation

Assumption	Hammering	Darts
Sample size	50 participants (5 000 observations)	50 participants (5 000 observations)
Effect of repetition (Calibration effect)	Between − 0.25 and − 1.25 mm	Between − 0.5 mm and − 2.5 mm
Residual SD/Variance	SD between 5 and 20 mm (Var between 25 and 400$\:{mm}^{2}$)	SD between 20 and 50 mm (Var between 400 and 2500$\:{mm}^{2})$
Effect of attempt (Learning effect)	10% of effect of repetition	10% of effect of repetition
Random variance	10% of residual variance	10% of residual variance
Model intercept	10 mm	50 mm

Please note that values in mm refer to deviations from the target

Results show that in each scenario, we can detect influences of the repetition as small as 0.75 mm in hammering and 1.5 mm in darts with a sufficient power of more than 80%. Moreover, in all scenarios, power is sufficient to detect effects of repetition as small as only one twentieth of the residual standard deviation.

### Participants

For this study, 50 participants were recruited, 12 identified as female, 37 as male, one as non-binary. Average age was 25.6 years (range 18–53 years, standard deviation 6.8 years). All data collected is reported with no incidence of missing or excluded data. Participants were pre-screened as “non-experts” based on a questionnaire filled out prior to the experiment. The questionnaire asked how often the participant performed each task, selecting from the following categories: “rarely/never” – 24 (darts) & 26 (hammer) participants, “yearly” – 16 (darts) & 22 (hammer) participants, “monthly” – 9 (darts) & 2 (hammer) participants, “weekly” – one (darts) & zero (hammer) participant, or daily – zero (darts) & zero (hammer) participants. The participants who answered “monthly” and “weekly” reported playing darts casually/recreationally in a bar setting. The two monthly hammer participants reported having recently remodeling projects at home. They were determined to be acceptable as “unskilled” given that they did not play darts competitively or performed hammering tasks as a profession and thus, did not engage within any kind of deliberate practice toward improving technical skill.

### Materials and procedures

The experimental set up included a paper target (A4 size paper) with a printed bullseye centered in 10 rings vertically mounted on a wall 2.5 m above the floor as well as a mark placed on the ground at 2.5 m distance from the wall for darts and 50 cm distance from the wall for hammering. The bullseye, a circle of 1 mm diameter, was used as the sole printed reference of measure to aim for during the task. Participants were instructed to remain behind the floor mark but were given the freedom to move anywhere laterally. Each task involved 20 rounds of 5 repetitions aiming for the bullseye (1 mm) of the target. At the completion of each set of repetitions, the participants were instructed to leave their position, for the darts to retrieve and for the hammering to step aside and look away. During this break, a researcher replaced the paper target with a new one and once safely clear, the participants returned to their position and repeated the task, total break time lasting about 30 s. A camera was set up to record the target during the duration of the experiment to enable the researcher to mark the order of throws/swings placed onto the target. A successful “hit” was considered when the dart/hammer punctured a hole into the paper target.

There were two tasks, darts and hammering with the order of tasks counterbalanced across participants. The darts were steel tipped, weighted darts commonly used in typical dart games with participants throwing 5 darts in succession per round, standing 2.5 m from the wall. The hammer was a geologist’s hammer which consisted of a steel head with a blunt end typical of normal hammers and a fine pointed end designed for chipping rocks as relevant for use in geology; no nails were used in the task. Participants were asked to swing the hammer five times aiming the pointed end at the target, with the blunt end pointed at, starting, and ending from the participant’s shoulder the whole time. Participants were instructed to swing confidently but not aggressively so that the pointed end puncturing a small (less than 1 mm) hole in the target during each swing without destroying the target or the wall. Subsequently, we will denote the five movements performed in immediate succession as *repetitions* and the twenty sets with five repetitions each as *rounds*.

Errors were measured based on the holes punctured in each paper target. If there was no hole punctured, the repetition was considered a miss. Each round consisted of one paper target with a new target exchanged in between each round. A camera pointed at the target recorded every hit on the target for the duration of the task in order to ascertain the order in which the hits occurred upon review of the video with each hit subsequently labeled on the target numerically.

### Data analysis

Each target was then scanned into a PDF file, and the errors were measured in millimeters as distances from the puncture points of the holes to the bullseye point on the target. Errors were measured horizontally and vertically along the X and Y axes with the bullseye representing the zero point via a specialized computer program written in Python that was utilized to ensure efficient and accurate measurements.

Based on the errors in both dimensions, the Euclidean distance of the puncture point from the bullseye was calculated. Subsequently, we will denote this overall distance (i.e. inaccuracy or error) as *distance* and the separate distances in x- and y- direction as *lateral distance* and *vertical distance* respectively. While the overall distance always is a positive value, lateral distance and vertical distance are signed errors and thus may be a negative (to the left in horizontal direction or downwards in vertical direction) or a positive (to the right in the horizontal direction or upwards in vertical direction) value depending on the direction of error made. Please note that some attempts in the darts task were inaccurate to such an extent that they totally missed the target space. We will denote these attempts as *missing* and have to exclude them from most of the further analyses, as no exact distance could be measured in these cases. To quantify and illustrate a possible calibration effect in the data, we report descriptive statistics and illustrate distances for both tasks and for each of the repetitions in each round separately.

Testing of the hypothesis (i.e. existence of calibration effect) requires careful consideration and separation of different effects that might influence the average distances in each attempt. Besides a hypothesized calibration effect in each round, participants might also show an improvement over the 100 attempts due to learning of the task, i.e. a general gradual improvement over time independent of the repetition number. Consequently, the statistical model is designed to identify the effect of calibration while controlling for gradual learning. Moreover, the model allows for the possibility of an interaction between these two effects, as learning might influence the first repetitions in each round more clearly than the later repetitions. Finally, participants might show a very heterogeneous ability to accurately perform the tasks, which should also be controlled for in the model. To take this nested structure of the data into account, the statistical model used is a linear mixed-effects model of the form:

$$\begin{array}{c}Distance=1+Attempt+Repetition+\\Attempt:Repetition+\left(1\:\vert\:Participant\right)\end{array}$$

Attempt (coded as 1–100) and Repetition (coded as 1–5) are fixed effects of this model, while the colon represents a possible interaction between both effects and the last term symbolizes a random effect, where we allow the intercept to vary by participant (see Brown, 2021, for more information on linear mixed-effect models and on the notation of the model, which is influenced by its implementation in R package lme4, Bates et al., 2015). Significance is tested by likelihood ratio tests performing a step-by-step comparison of the full model fit to restricted models of the same structure, but excluding the respective fixed effect (see Bates et al., 2015; Brown, 2021).

Moreover, we are interested in whether the error in a repetition actually depends on the error in the previous repetition and if yes, whether the participants over- or undercompensate for a previous error. Though reporting results of inferential statistics, we denote this as explorative analysis as we cannot theoretically derive a clear expectation and thus have not formulated a hypothesis on this. We limit ourselves to the first and second repetition, where the highest degree of calibration is to be expected. Again, we account for the nested structure of the data by fitting linear-mixed effects models of the form:

$$\:LateralDistance2=1+LateralDistance1+\left(1\:|\:Participant\right)$$

where LateralDistance2 (i.e. the error in x-direction in the second attempt) depends on LateralDistance1 (i.e. the error in x-direction in the first attempt) as a fixed effect and the intercept is allowed to vary by participant as a random effect. If participants fully compensate for the error in the first attempt, the error of the first attempt should not have an effect on the error in the second attempt, if participants over-compensate (under-compensate) for the error, we expect to see a negative (positive) effect of the error in the first attempt to the error in the second attempt. Significance is tested by a likelihood ratio test comparing the model fit to a restricted model of the same structure but excluding LateralDistance1. Additionally, the model is fitted for vertical errors (i.e. in y-direction) and for both errors in the hammering task with an analogue structure. All analyses were performed and visualized in R Statistical Software (v 4.3.1; R Core Team, 2023) using the packages *lme4* (Bates et al., 2015) and *ggplot2* (Wickham, 2016), among others. Data and material are available upon request from the corresponding author.

## Results

### Descriptive statistics and illustration

**Fig. 2:**
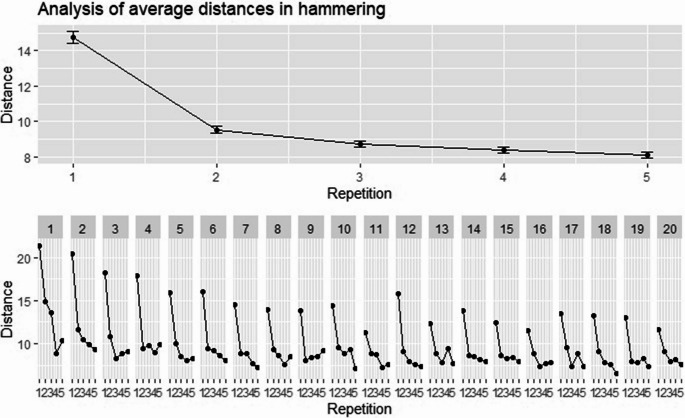
Analysis of the effects across repetitions in the hammer task. The upper part of the figure illustrates the overall average distances (mm) across repetitions 1 to 5, where error bars illustrate standard errors. The lower part of the figure illustrates the average distances per repetition further separated by rounds 1 to 20

**Fig. 3:**
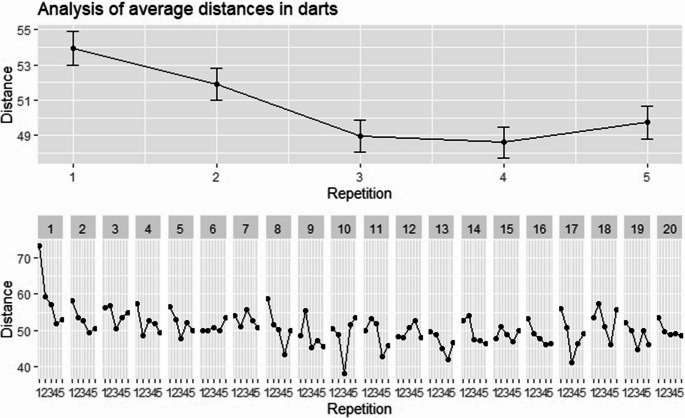
Analysis of the effects across repetitions in the darts task. The upper part of the figure illustrates the overall average distances (mm) across repetitions 1 to 5, where error bars illustrate standard errors. The lower part of the figure illustrates the average distances (mm) per repetition further separated by rounds 1 to 20

Figures 2 and 3 illustrate the average distances in millimeters (mm) across repetitions 1 to 5 overall as well as separated by round 1 to 20 for the hammer task and the darts task, respectively. Moreover, the descriptive statistics for repetitions 1 to 5 are summarized in Table 2. For hammering, the results suggest a gradual effect of calibration, most pronounced from the first to the second repetition, which is even clearly visible in each of the rounds. For darts, performance variations within each round are largely masked by noise due to the more difficult task, but overall results suggest calibration at least from the first to the second and the second to the third repetition. Please note that the fact that the calibration between second and third repetition appears even higher than between first and second, is very likely an artefact of the missing values, that is, of errors larger than the measurement range, being more prevalent in the first repetition. Moreover, it is important to note that our data include various observations per participant and thus the variation of distances (quantified as standard deviation in Table 2) is subject to both within- and between-subject variation. This nested structure will be accounted for by means of linear mixed-effects models.

**Table 2:** Descriptive statistics for the overall distances (mm) in repetitions 1 to 5 for the hammer and darts task respectively

	Distances hammer				
	1	2	3	4	5
Valid	1000	1000	1000	1000	1000
Missing	0	0	0	0	0
Mean	14.75	9.53	8.71	8.39	8.13
Std. Deviation	9.97	6.34	5.83	5.37	5.40
Minimum	0.66	0.36	0.51	0.49	0.00
Maximum	79.17	73.67	47.26	55.53	39.42
	Distances darts				
	1	2	3	4	5
Valid	864	879	893	888	885
Missing	136	121	107	112	115
Mean	53.93	51.90	48.94	48.62	49.74
Std. Deviation	27.70	27.30	27.09	25.96	27.24
Minimum	2.80	2.54	0.33	0.5	1.14
Maximum	150.66	148.34	140.89	159.41	137.96

### Hypothesis testing

Table 3 reports results of the linear mixed-effects model for the hammer as well as for the darts task. Coefficients for fixed effects are broadly comparable to and interpretable like the ones for a standard linear model without random effects, while the results for random effects provide an assessment of the between-participants variation in the data as well as the residual variation (i.e. the variation not explained by the model). For the fixed effects, all coefficients point in the expected direction. Coefficients for both repetition and attempt are negative indicating the hypothesized calibration effect as well as learning effects. Coefficients for interaction are positive, which is consistent with the idea of decreased calibration effects in later attempts.

**Table 3:** Results of fitting the linear mixed effects-model in relation to the hammer task based on *N* = 5,000 observations from 50 participants as well as in relation to the darts task with*N* = 4,409 observations from 50 participants

Hammer task			
Fixed effects			
	Estimate	Std. Error	
Intercept	18.11	0.62	
Attempt	−0.08	0.01	
Repetition	−2.14	0.12	
Attempt: Repetition	0.01	0.002	
Random effects			
Groups	Name	Variance	Std. Dev.
Participant	Intercept	11.26	3.36
Residual		35.80	5.98
Darts task			
Fixed effects			
	Estimate	Std. Error	
Intercept	59.19	2.32	
Attempt	−0.09	0.03	
Repetition	−1.59	0.54	
Attempt: Repetition	0.01	0.01	
Random effects			
Groups	Name	Variance	Std.Dev.
Participant	Intercept	112.7	10.61
Residual		620.8	24.92

When including repetition to a model already controlling for learning effects, the fit to the data significantly improves for both the hammer ($\:{\chi\:}^2\left(1\right)=515.16$, *p* <.001) and the darts task ($\:{\chi\:}^2\left(1\right)=14.94$, *p* <.001), being strong evidence for the existence of calibration effects and thus confirming the study’s hypothesis. We also find that the inclusion of learning effects significantly improves model fit (Hammering: $\:{\chi\:}^2\left(1\right)=158.76$, *p* <.001, Darts: $\:{\chi\:}^2\left(1\right)=22.84$, *p* <.001) and that an interaction effect significantly improves model fit for hammering ($\:{\chi\:}^2\left(1\right)=49.43$, *p* <.001) but not for darts ($\:{\chi\:}^2\left(1\right)=1.41$, *p* =.235). The insignificant interaction in darts might partly be attributable to the inherently higher difficulty of the task and noise in the data compared to the hammer task. In line with this reasoning, results are clearer in the hammer task, and the residual errors of the model are larger for the darts task. Even more complex model structures could allow the effects of attempt and repetition to vary by participant, thereby controlling for interpersonal differences in learning or calibrating. However, such a model structure was discarded as it led to issues with model singularity (indicating that the random structure of the model was too complex to be supported by the data) or to virtually unchanged results.

### Exploratory analyses

Table 4 summarizes results for the linear mixed-effects models for the hammer as well as for the darts task with the signed distance of the second repetition in both lateral (x-direction) and vertical (y-direction) directions as dependent variable. The focus here is on the effects of the signed distances in the first attempt. Coefficient estimates for these distances are positive for the hammer task, but inconclusive for the darts task. When testing for significance, we find that inclusion of the signed distance of the first attempt significantly improves the fit to the data for the hammer task (lateral distance: $\:{\chi\:}^2\left(1\right)=21.33$, *p* <.001; vertical distance: $\:{\chi\:}^2\left(1\right)=36.19$, *p* <.001), but not for the darts task (lateral distance: $\:{\chi\:}^2\left(1\right)=1.32$, *p* =.250; vertical distance: $\:{\chi\:}^2\left(1\right)=0.30$, *p* =.585). Again, a more complex random structure of the model had to be discarded. In summary, results show that, even if controlling for interpersonal differences, the error in the second attempt tends to be in the same direction as the error in the first attempt (though being smaller, as was evidenced before), yet only for the hammer task. This suggests that a bias in the correction for the initial error, if existing at all, rather tends towards under compensation and might be dependent on the exact task (see van Beers, 2009 for a similar finding).

**Table 4:** Results of the linear mixed effects-model in relation to the hammer task based on *N* = 1,000 observations from 50 participants as well as in relation to the darts task with *N* = 773 observations from 50 participants

Hammer task – Lateral distance			
Fixed effects			
	Estimate	Std. Error	
Intercept	0.43	0.38	
LateralDistance1	0.12	0.03	
Random effects			
Groups	Name	Variance	Std. Dev.
Participant	Intercept	4.97	2.23
Residual		45.78	6.77
Hammer task – Vertical distance			
Fixed effects			
	Estimate	Std. Error	
Intercept	−2.10	0.44	
VerticalDistance1	0.14	0.02	
Random effects			
Groups	Name	Variance	Std. Dev.
Participant	Intercept	4.56	2.14
Residual		58.28	7.63
Darts task – Lateral distance			
Fixed effects			
	Estimate	Std. Error	
Intercept	−1.52	2.10	
LateralDistance1	0.04	0.03	
Random effects			
Groups	Name	Variance	Std. Dev.
Participant	Intercept	130.7	11.43
Residual		1349.6	36.74
Darts task – Vertical distance			
Fixed effects			
	Estimate	Std. Error	
Intercept	−11.28	2.30	
VerticalDistance1	−0.02	0.03	
Random effects			
Groups	Name	Variance	Std. Dev.
Participant	Intercept	156.1	12.49
Residual		1597.4	39.97

## Discussion

This study sought to find evidence for the visuomotor calibration phenomenon explored in real-world data of experts by Wunderlich et al. (2020) within a controlled lab setting amongst non-skilled performers. For this, an experimental design consisting of two different precision-based tasks was set up to test whether the first repetition within a round of repeated attempts from a fixed position will be less accurate than the subsequent attempts within that same round.

It is important to point out that, except for e.g. van Beers (2009), previous research on visuomotor calibration focused on analyzing data that was sourced from existing competition results. This previous research found evidence for the calibration effect as it pertained to “real world” sport situations, such as competitive darts and basketball (Phatak et al., 2020; Wunderlich et al., 2020). Although previous research like van Beers (2009) are certainly related and show some comparable results, we consider the present research the first systematic demonstration of the calibration effect in a lab setting.

The present study serves to explore and identify evidence for visuomotor calibration within a controlled lab setting with non-expert participants. Furthermore, by setting up the experiment with two different tasks (darts and hammer), evidence for visuomotor calibration was both found and replicated also for a task of everyday life. As already mentioned in the introduction, related phenomena have been reported in the areas of sensorimotor adaptation, motor control, and motor learning as well as the PAPC model and the subsequent linear dynamical system of motor control (e.g., van Beers, 2009; van Beers et al., 2013; Redding & Wallace, 2006; Schmidt & Wrisberg, 2008)—and potentially sometimes the effect might have gone unnoticed due to the common practice of excluding the analyses of initial trials due to increased variability and prolonged reaction times. Nevertheless, we consider the present findings remarkable given the persistence of the calibration effect every time a series of trials is interrupted by a short break for both novices (present research) and experts (Phatak et al., 2020; Wunderlich et al., 2020).

Statistically, there are limitations by the study design and the model chosen. In terms of measurement in the darts task, attempts totally missing the target space had to be excluded from further analysis, affecting the distribution of the errors. As these extreme errors were most prevalent in repetition 1 (see Table 1), this might result in a slight underestimation of the true calibration effect for darts and potentially also influence the interaction effect for darts. Moreover, though already using a complex model accounting for the nested data structure, simplifications of the model compared to reality are present. Particularly, linearity in both calibration and learning can be considered a simplification, as both theoretical assumptions and the results found suggest a weakening of the effect over time.

Within the hammering task, the results are quite definite showing a clear pattern in the average distances of the first repetition compared to the subsequent repetitions. This is reflected in the overall data separated by repetition but also clearly visible in the data of each round and supported by the results of the linear mixed effects model accounting for the hierarchical data structure. It shows that there is a clear and statistically significant calibration effect, when controlling for a (likewise significant) learning effect. Also, the model indicates a statistically significant interaction between attempt and repetition, highlighting that the calibration effect slightly decreases with increased learning of the task. This result may be insightful when examined through the power law of learning in future research (Haider & Frensch, 2002).

Examining the darts data paints a similar picture. However, due to the non-expert participants, the darts task can be considered very difficult and was accompanied by high noise in the data as well as missing data because of too large errors. Visually, there is evidence indicating a calibration effect, even though it is not so cleanly displayed as for the hammering task. The results of the linear mixed model indicate significance for all fixed effects except for the interaction effect between attempt and repetition. Meaning that, through all the noise, there is a significant calibration effect identifiable even when accounting for a (likewise significant) learning effect and interpersonal differences. Within the later rounds, the calibration effect does appear to very slightly decrease in line with a learning effect, but the interaction effect did not indicate significance, which might partly be caused by the large noise in the darts distances and the necessity to exclude very large errors (misses).

This existence of a calibration effect might explain the anecdotal occurrence of initial “pretend performances” in tasks like hammering or throwing darts in which real performances (i.e., throwing a dart and hitting a nail with a hammer) are preceded by gentle (and partly visually controlled) movements without actually releasing the dart or hitting the nail. Given the present findings, it seems likely that people are (implicitly or explicitly) aware of the existence of the calibration effect and therefore sometimes execute the aforementioned pretending to help calibrate their performance before actually hitting a nail or throwing a dart.

The 20 rounds conducted of the darts and hammering task involved each participant physically stepping away from their position at the end of each round. This was necessary as part of our theoretical prediction regarding a “calibration reset” with respect to the relation between an internal and an external frame of reference, but it was also practical in regard to allowing the researcher to replace the paper target and for the participant to retrieve the darts following each round. As the effect appears to be most prominent, we focused solely on the first and second repetition of each round to exploratively analyze whether accuracy improvements actually depend on the previous attempt and whether participants show a tendency to over- or under-compensate for the previous error. Model results indicate dependence of the error in the second repetition on the error in the first repetition (in this case: under-compensation of the previous error), but only for hammering. Thus, our data show that, if controlling for interpersonal differences, the participants tended to under-compensate within the second attempt, but only in the hammering task. The tendency for over- or under-compensation should be further considered in future research with regard to task dependence and in comparison, between experts and non-experts in a similar experimental condition.

## Conclusion

In conclusion, the present findings provide evidence for the calibration effect in a sports (darts) and everyday task (hammering) in an experimental paradigm. This effect was present even when controlling for a (likewise significant) gradual learning effect. Moreover, the calibration effect tended to slightly decrease with increased learning, though only significant in the hammering task. Tests on whether participants tend to over-or under-compensate for the error in the first repetition gave inconclusive results, tending towards under-compensation for the hammering task, while not showing significant results in any direction for the darts task. Future study directions in this regard should consider exploring the calibration effect across different types of tasks (sport specific and everyday tasks), as well as among varying levels of expertise. This research may prove useful when considering the training and performance implications in sports like hockey, football, basketball, as well as accuracy sports such as darts, shooting, and archery.

## Data Availability

No datasets were generated or analysed during the current study.
